# Never Too Late: A Case Report of Severe Fanconi Syndrome Developing After More than a Decade of Silent Tenofovir Disoproxil Fumarate Exposure

**DOI:** 10.3390/reports9030244

**Published:** 2026-07-27

**Authors:** Vasileios Petrakis, Dimitrios Themelidis, Maria Panopoulou, Pelagia Kriki, Pipitsa N. Valsamaki, Dimitrios Papazoglou, Periklis Panagopoulos

**Affiliations:** 1 Department of Infectious Diseases, 2nd University Department of Internal Medicine, University General Hospital Alexandroupolis, Democritus University of Thrace, 68100 Alexandroupolis, Greece; 2 University Laboratory of Microbiology, University General Hospital Alexandroupolis, Democritus University of Thrace, 68100 Alexandroupolis, Greece; 3 University Department of Nephrology, University General Hospital Alexandroupolis, Democritus University of Thrace, 68100 Alexandroupolis, Greece; 4 Nuclear Medicine Department, Medical School, Democritus University of Thrace, 68100 Alexandroupolis, Greece

**Keywords:** tenofovir disoproxil fumarate, Fanconi syndrome, nephrotoxicity, proximal renal tubulopathy, HIV

## Abstract

**Background and Clinical Significance**: Tenofovir disoproxil fumarate (TDF) is a widely prescribed nucleotide reverse transcriptase inhibitor (NtRTI) for HIV-1 infection. Though generally well-tolerated, proximal renal tubulopathy resulting in full-blown Fanconi syndrome remains a rare but severe complication (<0.1%). **Case Presentation**: We present the case of a 52-year-old female living with HIV-1 (diagnosed in 1999, CDC stage A3) who had been treated with a TDF-based antiretroviral regimen for 12 years. Upon admission, she complained of progressive bone pain and polyuria over the preceding six months. Laboratory investigations revealed profound hypokalemia, severe hypophosphatemia, hypouricemia, elevated alkaline phosphatase (ALP) and a decline in renal function (creatinine 1.3 mg/dL from a baseline of 0.7 mg/dL). Arterial blood gas (ABG) analysis showed a normal anion gap hyperchloremic metabolic acidosis alongside respiratory acidosis. Urinalysis demonstrated profound glycosuria in the setting of normal blood glucose levels, coupled with increased 24 h urinary excretion of potassium and phosphorus. A bone scintigraphy demonstrated a “super scan” pattern of metabolic etiology, establishing secondary osteomalacia driven by renal phosphate wasting. Secondary hyperparathyroidism and severe vitamin D3 deficiency were also recorded. The diagnosis of TDF-induced Fanconi syndrome was established. TDF was discontinued, and her antiretroviral regimen was modified to tenofovir alafenamide fumarate (TAF), emtricitabine (FTC), darunavir, and ritonavir, combined with vitamin D supplementation. Over a 6-month follow-up period, renal function normalized, electrolyte wasting resolved, and metabolic acidosis completely reversed. **Conclusions**: This case highlights that TDF-induced proximal tubulopathy can manifest even after a decade of uneventful therapy, particularly when co-administered with a boosted protease inhibitor.

## 1. Introduction and Clinical Significance

The introduction of highly active antiretroviral therapy (HAART) has fundamentally shifted the clinical trajectory of human immunodeficiency virus type 1 (HIV-1) infection, turning a once-fatal disease into a manageable, chronic medical condition [1]. Among the cornerstone structural components of modern antiretroviral regimens, tenofovir has played a vital role due to its high efficacy, favourable resistance profile, and low rate of acute metabolic complications [2]. Historically administered as tenofovir disoproxil fumarate (TDF), this nucleotide reverse transcriptase inhibitor (NtRTI) has been widely used globally in both treatment-naive and treatment-experienced patients living with HIV [3]. 

Despite its extensive therapeutic success, clinical experience over the past decades has established a clear association between long-term TDF administration and specific structural or functional toxicities [4]. The most prominent among these involve the skeletal system, through a reduction in bone mineral density, and the renal system, through proximal tubular dysfunction [4]. TDF is a prodrug requiring initial conversion into its parent compound, tenofovir, which is subsequently phosphorylated to its active form within target host cells [5]. During this systemic circulation process, tenofovir is cleared significantly by the kidneys through a combination of simple glomerular filtration and active tubular secretion [6]. 

Active transport across the basolateral membrane of the proximal convoluted tubule cells is mediated by organic anion transporters, primarily OAT-1 and OAT-3 [6]. Once inside the tubule cells, the drug is pumped across the apical membrane into the urinary lumen via specialized efflux pumps known as multidrug resistance-associated proteins 2 and 4 (MRP-2 and MRP-4) [6]. A structural bottleneck or functional disruption at any stage of this pathway can cause intracellular accumulation of tenofovir [6]. Elevated intracellular concentrations exert a direct toxic effect on host mitochondrial DNA polymerase gamma, compromising mitochondrial replication, causing structural depletion of the respiratory chain, and halting ATP production [6]. Because the cells of the proximal convoluted tubule rely heavily on active transport mechanisms powered by mitochondrial ATP to reabsorb filtered solutes, this energetic failure disrupts multiple transport pathways [6]. 

When this dysfunction extends across all major transporters of the proximal tubule, it culminates in acquired Fanconi syndrome [7]. This rare clinical condition is characterized by a generalized failure to reabsorb glucose, amino acids, uric acid, potassium, bicarbonate, and inorganic phosphate from the ultrafiltrate [7,8]. While partial or mild proximal tubulopathies are noted more frequently, full-blown Fanconi syndrome remains a rare complication, occurring in less than 0.1% of people living with HIV who receive TDF-based antiretroviral therapy [9]. Typically, drug-induced Fanconi syndrome manifests relatively early during the therapeutic course, with most cases identified within the first one to twenty-nine months of exposure [10].

Developing full tubulopathy after more than a decade of stable, uneventful TDF therapy is an exceptional clinical phenomenon. In this report, we present the clinical details, laboratory profile, diagnostic process, and therapeutic resolution of a fifty-two-year-old female living with HIV who developed severe, late-onset TDF-induced Fanconi syndrome after twelve years of treatment. Her specific antiretroviral history, featuring a boosted protease inhibitor regimen, provides important insights into the drug–drug interactions that drive delayed mitochondrial toxicity within the renal cortex.

## 2. Case Presentation

### 2.1. Clinical Presentation and Patient Background

A fifty-two-year-old female patient with a long-standing history of HIV infection presented to the Emergency Department. The primary clinical reason for her acute admission was a confirmed positive result on both a rapid antigen test and a real-time reverse transcription polymerase chain reaction (RT-PCR) test for SARS-CoV-2. Given her underlying medical history and comorbidities, she met the criteria for high risk of severe COVID-19 progression, necessitating immediate hospitalization for the administration of a pre-emptive five-day course of intravenous remdesivir antiviral therapy. Beyond the diagnosis of HIV infection since 1999, the patient’s past medical history was notable for severe chronic obstructive pulmonary disease (COPD). She was a heavy smoker with a cumulative history of seventy pack-years, and her respiratory disease was characterized by frequent exacerbations that required recurrent hospitalizations for acute ventilatory support and medical stabilization.

Her chronological antiretroviral therapy history was highly complex and underscored the long-term clinical management of a treatment-experienced patient. In the year 2010, she officially initiated her first highly active antiretroviral therapy regimen, which consisted of a fixed-dose combination of tenofovir disoproxil fumarate, emtricitabine, and efavirenz. While this initial combination provided adequate virological suppression, the patient experienced severe central nervous system adverse effects, primarily manifesting as debilitating sleep disturbances and persistent insomnia, which directly compromised her quality of life. In the year 2011, a routine genotypic resistance assay was performed to map her resistance profile. The molecular sequencing results demonstrated complex viral mutations that conferred high-level resistance to several older nucleoside reverse transcriptase inhibitors and non-nucleoside reverse transcriptase inhibitors, alongside an intermediate resistance profile to tenofovir. To overcome these mutational barriers and maintain viral suppression, her antiretroviral regimen was modified. Her revised highly active antiretroviral therapy combination consisted of the protease inhibitor atazanavir, boosted with the metabolic enhancer ritonavir, combined with the nucleotide analogue tenofovir disoproxil fumarate and the integrase strand transfer inhibitor raltegravir. This tailored salvage regimen proved highly effective from an ideological standpoint, achieving stable, undetectable plasma HIV-1 RNA levels and a well-preserved CD4-positive T-lymphocyte count over the subsequent eleven years.

Upon her admission to the medical ward for COVID-19 management, a comprehensive clinical examination and systems review were performed. During this targeted review, the patient described a six-month history of progressive, generalized bone pain that primarily affected her axial skeleton, thoracic cage, shoulder girdles, and bilateral lower extremities. This pain had become progressively severe, limiting her mobility and hindering basic activities of daily living. Concurrently, she reported a pronounced onset of polyuria and compensatory polydipsia over the same six-month interval. She explicitly noted a substantial increase in her daily urine volume, which persisted throughout the night, disrupting her sleep independent of her previous medication-induced insomnia. Her subacute symptoms—specifically progressive bone pain, polyuria, and polydipsia—had been developing over the 6 months prior to admission and were unrelated to her acute SARS-CoV-2 infection. The COVID-19 diagnosis served as the catalyst for her emergency department presentation and hospitalization, enabling the formal diagnostic workup of her pre-existing Fanconi syndrome.

On initial physical evaluation, her vital signs were stable and within normal physiological boundaries. She was entirely afebrile, with a temperature of 36.6 °C. Her peripheral blood pressure was measured at 120/75 mmHg, and her resting heart rate was regular at 85 beats per minute. Her respiratory rate was 16 breaths per minute, and her peripheral oxygen saturation was measured at 96%. Neurologically, her sensorium was entirely clear (Glasgow Coma Scale score of 15/15), with no focal motor or sensory deficits. Cardiovascular auscultation revealed clear, rhythmic, and well-demarcated heart sounds, with no audible murmurs, rubs, or gallops. Her abdominal examination showed a soft, easily compressible, and completely non-tender abdomen, with normal active bowel sounds present in all four quadrants and no detectable hepatosplenomegaly. Her extremities were free of pitting lower-limb oedema, and her skin examination revealed no signs of acute exanthemata, petechiae, or peripheral track marks. Pulmonary auscultation revealed bilateral musical rales scattered across both lung fields, accompanied by a distinct, prolonged expiratory wheeze. These respiratory findings were consistent with her established baseline of chronic obstructive pulmonary disease, and she demonstrated no acute signs of respiratory distress, accessory muscle use, or cyanosis. A baseline twelve-lead electrocardiogram demonstrated normal sinus rhythm with normal intervals and no acute ST-segment or T-wave abnormalities. A portable anteroposterior chest radiograph revealed hyper-inflated lung fields and flattening of the diaphragmatic domes, typical of her emphysematous COPD history, but showed no signs of acute focal consolidations, interstitial infiltrates, or pleural effusions.

### 2.2. Laboratory Evaluation and Diagnostic Workup

To evaluate her subacute constitutional complaints of progressive bone pain and polyuria, a comprehensive biochemical profile was ordered upon admission. This assessment revealed major metabolic and renal deviations when cross-referenced with her historical annual laboratory data and data collected exactly one year prior. Her baseline haematological profile remained stable, with an admission haematocrit of 41.6% compared to 42.3% the previous year, a haemoglobin concentration of 13.2 g/dL, a total platelet count of 210,000/μL, and a total white blood cell count of 6190/μL with a normal differential distribution consisting of 56.7% polymorphonuclear neutrophils and 29.7% lymphocytes. Her serum glucose concentration on admission was completely normal at 98 mg/dL and the serum urea level was 27 mg/dL. However, her serum creatinine concentration demonstrated a clear, pathological increase, rising to 1.3 mg/dL from her previous stable baseline of 0.7 mg/dL. For over a decade preceding admission, her baseline serum creatinine remained stable at 0.7 mg/dL (eGFR > 90 mL/min/1.73 m^2^). The rising creatinine to 1.3 mg/dL on admission, coinciding with her 6-month history of symptoms, confirms that her proximal tubulopathy developed insidiously within the preceding 6 to 12 months. This elevation indicated an acute-on-chronic decline in her estimated glomerular filtration rate. Her hepatic enzyme profile showed normal transaminase activities, with a serum glutamic oxaloacetic transaminase (SGOT) of 19 U/L and a serum glutamic pyruvic transaminase (SGPT) of 12 U/L. Her gamma-glutamyl transferase (γ-GT) was also normal at 17 U/L. In stark contrast, her serum alkaline phosphatase (ALP) demonstrated an isolated elevation, rising sharply to 265 U/L from a baseline of 107 U/L the previous year (Table 1). Her serum lipid panel remained stable, with a total cholesterol of 245 mg/dL, a low-density lipoprotein (LDL) cholesterol of 135 mg/dL, and a high-density lipoprotein (HDL) cholesterol of 68 mg/dL. Her systemic inflammatory markers were unremarkable, with a C-reactive protein (CRP) level of 0.8 mg/dL.

Further analysis of her serum electrolyte and acid-base profile revealed severe abnormalities. While her serum sodium concentration was entirely normal at 140 mmol/L, her serum potassium level was low at 2.8 mmol/L (Table 1). This profound hypokalaemia occurred in the absence of gastrointestinal losses, as the patient explicitly denied any episodes of vomiting or diarrhoea. She also confirmed that she was completely compliant with her medical regimen and had not ingested any new medications, diuretics, or over-the- counter non-steroidal anti-inflammatory drugs. Due to the severe hypokalemia and associated risk of severe cardiac arrhythmias or QTc prolongation, the patient was immediately placed on continuous telemetry monitoring in the medical ward. Serial 12-lead electrocardiograms were performed, showing normal QTc intervals (420 ms) without U-waves or ventricular ectopy prior to prompt electrolyte repletion. During hospitalization, her baseline severe COPD was managed with inhaled tiotropium/olodaterol (2.5/2.5 µg, 2 puffs once daily) and inhaled budesonide/formoterol (160/4.5 µg, 2 puffs twice daily). Systemic corticosteroids and high-dose nebulized beta-agonists were avoided, ensuring that her hypokalemia was driven by renal tubular wasting rather than iatrogenic intracellular potassium shifts. 

Most notably, her biochemical analysis revealed severe hypophosphatemia, with a serum inorganic phosphorus concentration of 1.1 mg/dL. This was accompanied by severe hyperuricemia, with a serum uric acid concentration of 0.9 mg/dL compared to a historical level of 2.1 mg/dL (Table 1). To characterize this constellation of severe electrolyte imbalances, an arterial blood gas (ABG) analysis was performed. The results demonstrated a complex mixed acid-base disorder. Her systemic arterial pH was low at 7.25. Her partial pressure of carbon dioxide (pCO_2_) was elevated at 47 mmHg, indicating a primary or compensatory respiratory acidosis component stemming from her baseline chronic obstructive pulmonary disease. Her partial pressure of oxygen (pO_2_) was 72 mmHg, and her calculated serum bicarbonate (HCO_3_^−^) concentration was severely reduced at 20.1 mmol/L (Table 1). Her serum chloride concentration was elevated at 114 mmol/L. Using these values, her serum anion gap was calculated using the standard physiological formula: Serum Anion Gap = [Na^+^] − ([HCO_3_^−^] + [Cl^−^]) and was found 5.9 mEq/L. This value falls entirely within the normal physiological range, establishing the presence of a normal anion gap, hyperchloremic metabolic acidosis alongside her underlying respiratory acidosis. A 24 h urine collection upon admission confirmed true polyuria with a total volume of 3800 mL/24 h.

To localize the site of this profound solute and bicarbonate wasting, a detailed spot urinalysis and a concurrent twenty-four-hour urine collection were performed. Her spot urinalysis revealed a urine pH of 7.0, which was inappropriately alkaline given her systemic metabolic acidosis, a finding highly suggestive of a proximal tubule defect in bicarbonate reabsorption (Table 1). Her urine specific gravity was 1.015, and the biochemical dipstick analysis was entirely negative for protein, ketones, bilirubin, urobilinogen, and nitrites. Crucially, the urinalysis revealed heavy, macroscopically visible glycosuria. Because her concurrent serum glucose concentration was normal at 98 mg/dL, this heavy excretion of glucose into the urine represented a complete failure of renal tubular glucose reabsorption rather than an over-filtration secondary to systemic hyperglycemia. Furthermore, the 24 h urine collection confirmed a massive increase in the fractional excretion of both potassium and inorganic phosphorus, confirming that her profound hypokalaemia and hypophosphatemia were driven by ongoing, inappropriate renal wasting. Urinalysis dipstick showed no overt protein, and a quantitative 24 h urinary protein excretion was 0.35 g/24 h. The absence of nephrotic-range proteinuria (<0.5 g/24 h) helped rule out primary glomerular lesions such as HIV-associated nephropathy (HIVAN) or immune-complex glomerulonephritis, further confirming an isolated proximal tubular insult. Given the severe hypophosphatemia, elevated alkaline phosphatase, and chronic bone pain, further testing was conducted to assess bone mineral metabolism and skeletal structure. Her serum parathyroid hormone (PTH) concentration was elevated at 80 ng/dL, and her serum twenty-five-hydroxyvitamin D3 level was low at 14 ng/mL. Her total serum calcium concentration remained within normal physiological limits. Serum total calcium on admission was 8.9 mg/dL (corrected calcium: 9.1 mg/dL; reference range: 8.5–10.2 mg/dL). Despite severe vitamin D deficiency (14 ng/mL) and profound phosphate wasting (1.1 mg/dL), normocalcemia was preserved via secondary hyperparathyroidism (PTH 80 ng/dL), which enhanced bone resorption (Table 1). 

**Figure 1:**
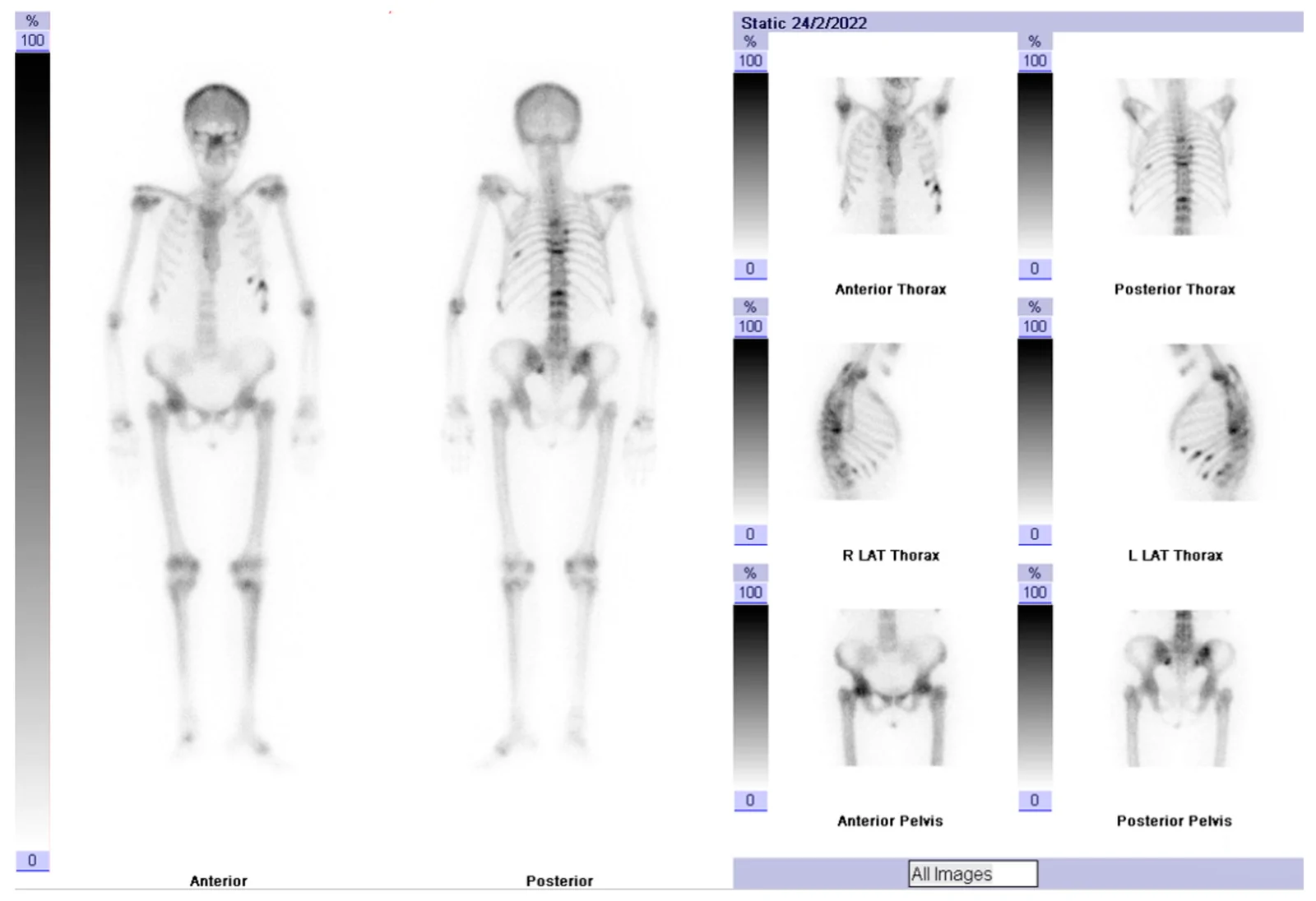
Whole-body ^99m^Tc-MDP bone scintigraphy and targeted joint views revealing a classic metabolic “super scan” pattern secondary to chronic renal phosphate wasting.

To evaluate the structural consequences of her long-standing phosphate wasting, a technetium-ninety-nine-m (^99m^Tc) methylene diphosphonate whole-body nuclear bone scintigraphy was performed at the Nuclear Medicine Department. The imaging revealed a classic, highly pathological metabolic “super scan” pattern (Figure 1). There was a symmetric, intense, and diffuse increase in radiotracer uptake throughout her entire calvarium, both shoulder joints, bilateral elbow joints, wrists, bilateral hip joints, sacroiliac articulations, and both knee joints, alongside extensive uptake across the lower bilateral rib cages. This dramatic imaging presentation provided diagnostic confirmation of widespread, high-turnover metabolic osteomalacia secondary to chronic, severe renal phosphate wasting. While specialized novel tubular markers (such as urinary β2-microglobulin) were not processed, the combination of marked polyuria, glycosuria, normal anion gap metabolic acidosis, and 99mTc-MDP bone scintigraphy provided unequivocal quantitative evidence of severe tubulopathy and metabolic osteomalacia. 

### 2.3. Final Diagnosis and Therapeutic Intervention

Integrating her clinical presentation of bone pain and polyuria with her laboratory findings—including hyperchloremic metabolic acidosis, a normal serum anion gap, heavy glycosuria with normal blood glucose, renal potassium and phosphate wasting, hyperuricemia, secondary hyperparathyroidism, and metabolic super scan imaging—the diagnosis of full-blown acquired Fanconi syndrome was established. Given her twelve-year history of continuous nucleotide analogue therapy, the condition was attributed to tenofovir disoproxil fumarate-induced proximal renal tubulopathy.

The primary therapeutic intervention required the immediate cessation of the offending nephrotoxic agent. However, because the patient possessed an intermediate resistance profile to tenofovir and high-level resistance to several older nucleoside analogues, maintaining optimal virological suppression required careful management. To protect her renal cortex while preserving viral control, her highly active antiretroviral therapy regimen was updated. Tenofovir disoproxil fumarate was permanently discontinued and replaced with tenofovir alafenamide fumarate (TAF), maintained in combination with emtricitabine, darunavir, and ritonavir. Tenofovir-free regimens—such as dual-therapy options (e.g., dolutegravir/lamivudine or boosted darunavir + lamivudine)—were evaluated. However, given her historical multi-class NNRTI/NRTI resistance profile, retaining TAF within a robust boosted protease inhibitor regimen was necessary to secure viral suppression. Unlike TDF, which requires dose interval adjustments when CrCl falls below 50 mL/min, TAF requires no dosage adjustment in patients with an estimated CrCl ≥ 15 mL/min (or in patients on chronic hemodialysis) [7]. Standard dosing of fixed-dose TAF/FTC (25/200 mg once daily) was therefore safely utilized.

Concurrently, medical repletion was initiated, consisting of high-dose oral vitamin D3 supplementation and oral electrolyte replacements tailored to correct her potassium and phosphate deficits. Initial medical repletion consisted of oral potassium chloride (40 mEq/day), oral neutral sodium-potassium phosphate (providing approximately 1000 mg of elemental phosphorus daily in divided doses), and high-dose oral cholecalciferol (50,000 IU weekly for 8 weeks), adjusted based on serial lab monitoring until full resolution.

### 2.4. Clinical Course and Follow-Up

Following the therapeutic switch from TDF to TAF and the initiation of electrolyte repletion, the patient demonstrated a prompt and steady improvement in both her clinical symptoms and biochemical markers. Her polyuria and polydipsia began to subside within the first two weeks of treatment, and her generalized bone pain gradually resolved over the subsequent months, restoring her physical function to her baseline status.

The long-term recovery of her metabolic, renal, and acid-base parameters was monitored through systematic follow-up evaluations at two months and six months post-intervention (Table 1). At her two-month follow-up, her serum creatinine had decreased to 1.12 mg/dL, and her serum alkaline phosphatase dropped to 235 U/L. Her serum potassium rose into a safer range at 3.4 mmol/L, her serum phosphate increased to 1.4 mg/dL, and her serum uric acid rose to 1.7 mg/dL. Her serum chloride had dropped to 108 mmol/L, and her vitamin D3 level rose to 28 ng/mL. Her spot urinalysis at two months demonstrated a lower urine pH of 6.5, a urine specific gravity of 1.009, and only trace amounts of glucose remaining in her urine. Her arterial blood gas profile showed a resolving pH of 7.32, with an increased serum bicarbonate concentration of 23.4 mmol/L.

**Figure 2:**
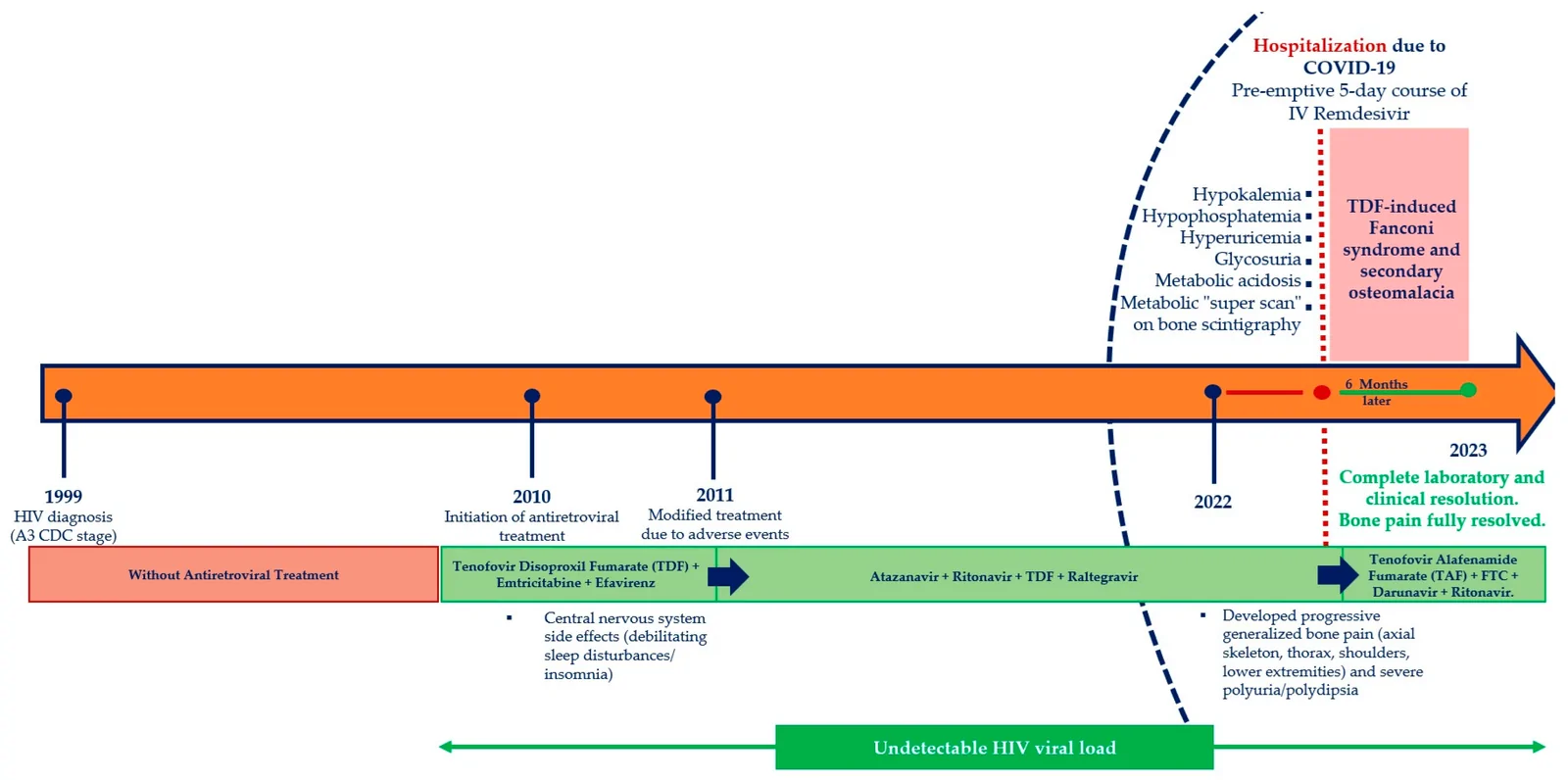
Clinical timeline illustrating the patient’s medical history from initial HIV diagnosis through the development and successful management of Tenofovir Disoproxil Fumarate (TDF)-induced Fanconi syndrome.

By her six-month follow-up evaluation, the patient achieved complete laboratory and clinical resolution of her proximal tubulopathy. Her serum creatinine had fully normalized to 0.91 mg/dL, returning to her pre-injury baseline. Her serum alkaline phosphatase fell into the normal reference range at 195 U/L. Her serum electrolyte targets were fully achieved, with her serum potassium stable at 3.8 mmol/L, her serum phosphate normalized to 1.9 mg/dL, and her serum uric acid restored to 2.1 mg/dL. Her serum chloride fell to a normal level of 102 mmol/L, and her vitamin D3 levels reached an optimal concentration of 46 ng/mL. Crucially, her spot urinalysis at six months demonstrated a normal urine pH of 6.0, a urine specific gravity of 1.010, and a complete absence of glycosuria. Her arterial blood gas profile demonstrated full resolution of her normal anion gap metabolic acidosis, with a systemic pH of 7.36 and a normal serum bicarbonate concentration of 26.4 mmol/L. Throughout this six-month follow-up period, her plasma HIV-1 RNA remained completely undetectable, confirming that the switch to a TAF-based regimen effectively protected her renal function without compromising her long-term virological control (Figure 2). At the follow-up evaluations at 2 and 6 months, the HIV-RNA viral load was undetectable and the CD4 cell count remained stable (458 and 466 cells/mm^3^, respectively) (Table 1). 

## 3. Discussion

This case highlights a key clinical principle: late-onset drug toxicity can manifest even after more than a decade of stable, uneventful therapy. While tenofovir disoproxil fumarate-associated Fanconi syndrome is a well-documented clinical entity, it typically presents within the first few years of drug exposure [11]. The development of a full proximal tubulopathy after twelve years of continuous TDF treatment represents a unique clinical trajectory that underscores the cumulative nature of antiretroviral-induced mitochondrial damage within the renal architecture [12]. The underlying pathophysiology of TDF-induced Fanconi syndrome centres on the transport mechanics of the proximal convoluted tubule cells within the renal cortex [12]. Tenofovir is a small, hydrophilic molecule that relies on specialized transporter networks for its elimination [11]. It enters the proximal tubule cell from the vasa recta via the basolateral organic anion transporters OAT-1 and OAT-3 [11]. Under normal physiological conditions, the drug is then efficiently pumped out of the cell and into the urinary space across the apical membrane via the ATP-dependent efflux pumps MRP-2 and MRP-4 [11].

However, the risk of long-term toxicity is significantly increased by specific drug–drug interactions within this transporter network [13]. Our patient’s highly active antiretroviral therapy regimen since the year 2011 included the protease inhibitor atazanavir, boosted with the metabolic enhancer ritonavir. Pharmacokinetic and clinical data indicate that ritonavir-boosted protease inhibitors can directly inhibit the apical MRP-4 efflux pumps [14]. This creates a structural bottleneck, allowing tenofovir to enter the cell via basolateral OAT transporters but blocking its apical exit into the urine [14]. Over an extended period, this mismatch results in a high intracellular concentration of the drug within the proximal tubule cells [14].

Once accumulated inside the cytoplasm, tenofovir exerts a direct toxic effect on host cell mitochondria [15]. It competitively inhibits mitochondrial DNA polymerase gamma, leading to the depletion of mitochondrial DNA, structural alterations in the mitochondrial respiratory chain, and a profound failure of cellular ATP production [15]. Because the proximal convoluted tubule is the most metabolically active segment of the nephron, its multiple symporters and antiporters rely entirely on ATP-driven sodium-potassium ATPase pumps to reabsorb solutes against steep concentration gradients [16]. When mitochondrial ATP production is halted by chronic tenofovir toxicity, these active transport systems collapse simultaneously [16]. This widespread transport failure leads to the clinical presentation of Fanconi syndrome, characterized by the inappropriate urinary wasting of vital solutes, including glucose, amino acids, potassium, uric acid, and inorganic phosphate [17]. The diagnosis of Fanconi syndrome was directly evidenced in our patient by the triad of heavy glycosuria in the presence of normal blood glucose (98 mg/dL), severe hypokalemia (2.8 mmol/L), and normal anion gap hyperchloremic metabolic acidosis (pH 7.25, HCO3^−^ 20.1 mmol/L). Furthermore, the chronic phosphate wasting (serum phosphorus 1.1 mg/dL) directly precipitated secondary osteomalacia, evidenced clinically by severe bone pain and biochemically by an isolated ALP elevation (265 U/L) and secondary hyperparathyroidism (PTH 80 ng/dL). These findings correlate precisely with established literature demonstrating that long-term TDF accumulation disrupts proximal tubule ATP production, leading to multi-solute transport breakdown [14,15,16,17].

The structural consequences of long-standing proximal tubulopathy are vividly illustrated by our patient’s musculoskeletal presentation. The ongoing wasting of filtered phosphate leads to chronic hypophosphatemia, which prevents the proper mineralization of osteoid tissue throughout the skeletal system [18]. In adults, this presents as metabolic osteomalacia, characterized by severe, progressive bone pain and a high risk of insufficiency fractures [19]. The extensive skeletal remodeling driven by chronic phosphate wasting triggers a robust osteoblast response, which explains the isolated elevation of serum alkaline phosphatase observed upon her admission. This high-turnover bone pathology was clearly captured by her whole-body nuclear scintigraphy, which demonstrated a classic metabolic “super scan” pattern. This image is characterized by an intense, diffuse, and symmetric radiotracer uptake across the entire skeleton, with minimal or absent radiotracer excretion visible within the renal parenchyma, a direct reflection of her profound metabolic bone disease.

The definitive resolution of this severe metabolic crisis was achieved by replacing tenofovir disoproxil fumarate with tenofovir alafenamide fumarate. TAF is a newer lipophilic prodrug of tenofovir that possesses distinct pharmacokinetic advantages over its older TDF counterpart [20]. TAF remains highly stable within the systemic plasma circulation, undergoing rapid intracellular cleavage to active tenofovir primarily after entering its target lymphoid cells and HIV-susceptible mononuclear cells [21]. Consequently, TAF administration maintains high antiviral efficacy while reducing circulating plasma tenofovir concentrations by approximately 90% [21]. This reduction drastically lowers the exposure of basolateral renal OAT transporters to the drug, shielding the proximal tubule cells from intracellular drug accumulation and subsequent mitochondrial damage [21]. As demonstrated in our patient’s clinical course, switching to a TAF-based regimen halts ongoing cellular toxicity, allowing the proximal renal tubules to structurally and functionally recover [22]. This led to the complete cessation of urinary solute wasting, the normalization of her serum creatinine, and the full resolution of her metabolic acidosis and metabolic bone disease within a six-month interval. However, from a global health perspective, cost-effectiveness remains a crucial consideration. In resource-limited settings where generic TDF is widely utilized due to low cost, universal switching to TAF may not be financially feasible [22]. In such environments, routine monitoring of dipstick glycosuria (in euglycemic patients) and serum creatinine offers a low-cost screening strategy to detect early tubular injury before full Fanconi syndrome develops [14,15,16,17,18]. When TDF toxicity occurs, alternative lower-cost generic tenofovir-free backbones (such as dual-therapy regimens) should be evaluated based on resistance history if TAF access is restricted [14,15,16,17,21,22].

## 4. Conclusions

In conclusion, this case provides a clear demonstration that tenofovir disoproxil fumarate-induced proximal renal tubulopathy can manifest as a delayed, late-onset complication after more than a decade of stable therapy. Clinicians must maintain long-term vigilance when managing patients on TDF-based antiretroviral therapy, particularly when co-administered with ritonavir-boosted protease inhibitors known to disrupt apical drug efflux mechanisms. The presence of a normal blood glucose level in the setting of heavy glycosuria remains a critical diagnostic indicator for proximal tubular dysfunction. When severe, the resulting chronic phosphate wasting can lead to metabolic osteomalacia, visible as a characteristic “super scan” on nuclear imaging. Finally, our patient’s excellent long-term recovery demonstrates that switching from TDF to a TAF-based antiretroviral regimen effectively prevents further nephrotoxicity and allows for complete functional renal and metabolic recovery while maintaining optimal virological suppression.

## Figures and Tables

**Table 1:** Longitudinal biochemical and acid-base parameters at diagnosis and following therapeutic switch from Tenofovir Disoproxil Fumarate (TDF)- to Tenofovir Alafenamide Fumarate (TAF)-based antiretroviral regimen.

Laboratory Examination		Unit of Measurement	At Diagnosis	After Switch from TDF to TAF-Based Antiretroviral Treatment	
			Value	At 2 Months	At 6 Months
Serum blood laboratory examinations	Glucose	mg/dL	98	112	104
	Urea	mg/dL	27	26	23
	Creatinine	mg/dL	1.3	1.1	0.9
	Alkaline phosphatase	U/L	265	235	195
	Potassium	mmol/L	2.8	3.4	3.8
	Uric acid	mmol/L	0.9	1.4	1.9
	Phosphorus	mmol/L	1.1	1.7	2.1
	Chloride	mmol/L	114	108	102
	Vitamin D	ng/mL	14	28	46
	Parathyroid Hormone	ng/dL	80		43
HIV specific data	CD4 cell count	Cells/mm^3^	423	458	466
	HIV-RNA	Copies/mL	Undetectable	Undetectable	Undetectable
Urinary laboratory examinations	pH		7	6.5	6
	Specific Gravity		1.015	1.009	1.010
	Glucose		(+++)	(−)	(−)
Arterial Blood Gas Analysis	pH		7.25	7.32	7.36
	PO_2_	mm Hg	72	69	73
	PCO_2_	mm Hg	47	46	46
	Potassium	mmol/L	2.3	3.2	3.6
	Lactate acid	mmol/L	0.3	0.4	0.3
	HCO_3_	mmol/L	20.1	23.4	26.4

## Data Availability

The research data are available after applying to the corresponding author due to privacy concerns.
